# Seed Predation by the Shore Crab *Carcinus maenas*: A Positive Feedback Preventing Eelgrass Recovery?

**DOI:** 10.1371/journal.pone.0168128

**Published:** 2016-12-15

**Authors:** Eduardo Infantes, Caroline Crouzy, Per-Olav Moksnes

**Affiliations:** Department of Marine Sciences, University of Gothenburg, Gothenburg, Sweden; Helmholtz-Zentrum fur Ozeanforschung Kiel, GERMANY

## Abstract

There is an increasing interest to restore the ecosystem services that eelgrass provides, after their continuous worldwide decline. Most attempts to restore eelgrass using seeds are challenged by very high seed losses and the reasons for these losses are not all clear. We assess the impact of predation on seed loss and eelgrass establishment, and explore methods to decrease seed loss during restoration in the Swedish northwest coast. In a laboratory study we identified three previously undescribed seed predators, the shore crab *Carcinus maenas*, the hermit crab *Pagurus bernhardus* and the sea urchin *Strongylocentrotus droebachiensis*, of which shore crabs consumed 2–7 times more seeds than the other two species. The importance of shore crabs as seed predators was supported in field cage experiments where one enclosed crab caused 73% loss of seeds over a 1-week period on average (~ 21 seeds per day). Seedling establishment was significantly higher (14%) in cages that excluded predators over an 8-month period than in uncaged plots and cages that allowed predators but prevented seed-transport (0.5%), suggesting that seed predation constitutes a major source of seed loss in the study area. Burying the seeds 2 cm below the sediment surface prevented seed predation in the laboratory and decreased predation in the field, constituting a way to decrease seed loss during restoration. Shore crabs may act as a key feedback mechanism that prevent the return of eelgrass both by direct consumption of eelgrass seeds and as a predator of algal mesograzers, allowing algal mats to overgrow eelgrass beds. This shore crab feedback mechanism could become self-generating by promoting the growth of its own nursery habitat (algal mats) and by decreasing the nursery habitat (seagrass meadow) of its dominant predator (cod). This double feedback-loop is supported by a strong increase of shore crab abundance in the last decades and may partly explain the regime shift in vegetation observed along the Swedish west coast.

## Introduction

Coastal ecosystem degradation is occurring worldwide as a result of anthropogenic activities [1]. One of the world’s most threatened ecosystems is seagrass habitats which are disappearing in all parts of the world at an alarming rate. It has been estimated that nearly 30% of the global seagrass area has been lost since the early 1900s, with an accelerating loss rate [2–4]. In many parts of the world, nutrient pollution and coastal eutrophication are considered a major cause of seagrass decline, where seagrass beds become dominated or replaced by mats of ephemeral macroalgae [5,6]. However, many seagrass systems have failed to recover even though nutrient loads have decreased, possibly because shifts in environmental conditions are generating feedback mechanisms that maintain the system in an unvegetated or algal dominated state [7–10]. These types of regime shifts [11] appear to be particularly common in shallow coastal ecosystems where increased sediment resuspension [12,13] and local recycling of sediment nutrients [7] are suggested as positive feedback mechanisms that prevent the return of seagrass.

Changes in the coastal food webs caused by overfishing of large fish predators can contribute to seagrass decline through a trophic cascade. For example, a decline in large predators increase mesopredators (e.g., small fish and decapod predators) and in turn decrease the abundance of algae mesograzers (e.g., small crustaceans and gastropods), thereby releasing ephemeral algae from grazer control negatively affecting seagrass [14–16]. Since large fish lack nursery habitats when seagrass beds decline, such cascading effects can create a feedback loop resulting in an accelerating loss of seagrass [17]. Most of existing research has focused on indirect top-down effects from mesopredators on seagrass. Less is known about direct effects of omnivorous mesopredators that may consume seagrass shoots or seeds.

Eelgrass (*Zostera marina* L.) is one of the most widely distributed species of seagrass and is the dominating species of the temperate North Atlantic [4]. As a result of anthropogenic impact, eelgrass has experienced rapid decline throughout is distributional range, including northern Europe and Scandinavia [3,18]. On the Swedish west coast, around 60% of the eelgrass has been lost since the 1980s [19]. Studies suggest that the primary mechanism behind the decline is an increased abundance of ephemeral algal mats that cover the eelgrass beds during the summer, caused by eutrophication in combination with overfishing, which has caused a trophic cascade that promote the growth of algae [20–22]. Despite decreasing nutrient loads to the coastal waters, no recovery of eelgrass has occurred [23,24]. Since eelgrass meadows provide a number of important functions to the coastal systems, as well as several valuable ecosystem services to society [25,26], there is an increasing interest to develop restoration techniques to recover these habitats in Scandinavian waters. Recent studies to develop restoration methods using seeds along the Swedish west coast found very high seed losses, making it difficult to use seeds for large scale restoration due to the low seedling establishment [27,28]. The results indicate that the seed losses may be due to predation [28], but eelgrass seed predators have never been identified in this area. In Sweden, seeds are released from the reproductive shoots in July-August and remain dormant in the sediment until the spring, when they germinate. During this long dormancy period of 7–9 months, seeds are exposed to transport by hydrodynamics and predators, and likely also suffer high mortality due to infections and premature germination [28].

Predation of eelgrass seeds has been documented in some parts of the world where several species of crabs, mollusks, fish, turtles and ducks have been identified as predators [29–33]. However, little is known about potential seed predators and their impact on eelgrass in northern Europe, since none of these previously reported seed predator species is present in this region. One potential seed predator in Europe is the shore crab *Carcinus maenas*. The shore crab is an opportunistic omnivore that feeds on various benthic organisms including infauna, mobile epifauna and plant material [34,35] Recent studies have found that shore crabs can consume parts of eelgrass shoots [36], but little is known regarding its capacity for seed predation. The shore crab is of special interest since it is a very abundant species in northern Europe and has shown a dramatic increase in abundance in the last decades as the populations of cod has decreased [37,38].

The aim of this study is to a) identify species that can prey on eelgrass seeds, b) assess if seed predation is a major cause of the low seedling establishment observed along the west coast of Sweden, c) assess if seed burial can reduce seed losses and increase seedling establishment. We also describe new positive feedback mechanisms that could explain observed changes in coastal vegetation and lack of eelgrass recovery in the study area.

## Methods

### *Zostera marina* seed predators

To identify eelgrass seed predators commonly found in eelgrass meadows in Sweden, a total of nine invertebrate predators and omnivores was studied in the laboratory. The dominating size-ranges of the predators found in the field when eelgrass seeds are released were included in the study (Table 1). Eelgrass seeds were collected by harvesting reproductive shoots in the Gullmars fjord, Gåsö at 1–3 m depth on 18-Jul 2014. Reproductive shoots were stored in outdoor tanks at the Sven Lovén Center, Kristineberg station until the seeds were released. Permission to harvest eelgrass shoots and for carrying out field experiments were obtained from the Swedish Administrative Board of Västra Götaland. Animal species were collected in an eelgrass meadow at Bökevik bay, near Kristineberg. Collected animals were measured and weighted, and used within 3 days of collection. Animals were starved for 24 h prior to experimentation. Seed sizes were 1.5 x 3.0 mm diameter and a weight of 7 mg.

**Table 1:** Seed predation in the laboratory. Tested sizes for each species (carapace width for *C*. *maenas and P*. *bernhardus*, total length for *Palaemon sp*., shell length for other species) and biomass (wet weight). Seeds consumed after 24 h, mean+SE, (10 seeds offered).

Animal	Species	Size (cm)	Biomass (g)	Replicates	Predation (%)
Decapod	*Carcinus maenas*	1.1–6.4	0.4–65.7	30	51.5 ± 7.5
	*Pagurus bernhardus*	0.5–1.4	0.2–1.3 (no shell)	25	6 ± 2.5
	*Palaemon elegans*	2.2–4.1	0.64	25	0
	*Palaemon adspersus*	2.5–5.5	0.23–0.70	25	0
Echinoderm	*Strongylocentrotus droebachiensis*	1.2–2.8	N/A	20	36.7 ± 9.3
	*Asterias rubens*	5–21	5–42	25	0
Gastropod	*Hinia nitida*	1.2–2.8	N/A	25	0
	*Littorina littorea*	1.7–3.5	N/A	25	0
	*Rissoa membranacea*	0.2–0.7	N/A	25	0

The experiment was carried out in 15 PVC tanks (36 x 26 x 22 cm) with flow-through surface water from the fjord to maintain similar conditions as in the field. Ten eelgrass seeds were placed on the bottom of the tanks after one randomly chosen animal was placed in each tank. Trials were run for 24 hr and the setup repeated during a two-month period (Jul and Aug of 2014) using 20–30 replicates per species (Table 1). After each trial, the number of remaining seeds was counted and the percentage of lost seeds was estimated for each species. Damaged and broken seeds were included as losses since seed viability was already lost.

### Burial depth and predation

To assess if burial protected seeds from predation, ten seeds were placed either at the sediment surface, at 1 cm or at 2 cm depth in separate 24 hour trials. Three predators of eelgrass seeds identified in the first study (shore crabs, hermit crabs and sea urchins) were tested. The experiment was carried out in the tanks used in the first study, provided with a 5 cm layer of natural sediments collected in a nearby bay. The sediment was pre-sieved (1 mm) to remove potential eelgrass seeds and debris, shells, or organisms that could affect the experiment. One animal was placed per tank with 15 replicates of each burial depth. After each trial, the sediment was sieved through a 1 mm mesh to recover the seeds. Pilot studies without predators showed that 100% of the seeds were recovered with this method (n = 10).

Although the gastropod *Hinia nitida* did not consume any seeds in the first experiment, pilot studies indicated that these snails could bury the seeds below the sediment surface, which may affect rates of predation and transport. To assess the prevalence of this behavior, we included an extra treatment with 10 seeds placed on the surface, on 5 replicated tanks with 6 *H*. *nitida* snails per tank (representing natural field densities; approximately 70 snails m^-2^). After 24 hours, seed burial by snails was calculated as the number of seeds that were not visible in the sediment surface.

### Seed predation in the field: 1-week experiment

**Fig 1:**
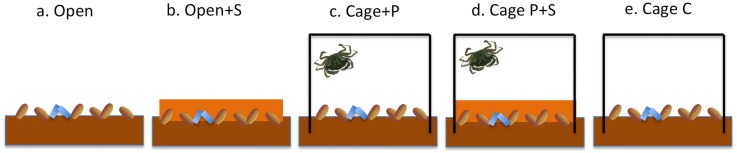
Diagram of treatments used in the 1-week field experiment. a) seeds on the sediment surface (Open), b) seeds covered with sand (Open+S), c) cage with seeds and a shore crab (Cage+P), d) cage with seeds covered with sand and a shore crab (Cage P+S) and e) cage with seeds (Cage C). Seed mimics represented in blue. The figure is not drawn to scale.

A cage experiment was designed to assess the effect of predation and transport on seed loss in the field, and to test whether or not covering the seeds with sediment would reduce loss rates. To assess the effect of transport of seeds by waves and currents, non-edible, artificial seed mimics were also used. Five cage treatments were used: a) uncaged seeds on the sediment surface (Open), b) uncaged seeds covered with sand (Open+S), c) closed cage with seeds on the sediment surface and a shore crab (Cage+P), d) closed cage with seeds covered with sand and a crab (Cage P+S) and e) closed cage with only seeds on the sediment surface (Cage C; Fig 1). In the sand-cover treatments, seeds were covered with a 2 cm layer of sieved (2 mm) natural sand collected in the same bay. Shore crabs between 20–40 mm were selected as seed predators for the cage experiment since this species and size showed the highest seed predation rates in the laboratory (see results). One shore crab was used in each cage, equivalent to 5 crabs m^-2^, which is representative of high natural densities in shallow bays in the study area [39,40].

The experiment was carried out in a semi-exposed bay (Bökevik) with sandy sediment and a natural eelgrass bed in August 2014, which coincides with the period when eelgrass seeds are naturally released from reproductive shoots along the Swedish west coast (E. Infantes, *unpubl*. *data*). The experimental plots (0.2 m^2^) were placed at 1.5 m depth, 5 m apart, along a transect parallel to the coast, at least 5 m from the nearest natural eelgrass patch. The plot corners were marked with nailed ribbons for later identification. Cages were made of a 1 mm nylon mesh (Sefar-nitex 06-1000/57) measuring 0.45 x 0.45 x 0.60 m, which excludes all potential seed predators. The lower 10 cm of the cages were buried in the sediment and the tops were closed using cable ties. Previous studies in shallow bays in the study area have found no detectable effects of cage artifacts (such as reduced light and water flow caused by the cage structure) on the benthic community after 3 and 6 weeks when using these cages [20]. In the study, no significant differences were found between treatments of cages with holes (open cages) and without cages for any biological variable [20]. These cages also allow small epibenthic fauna to migrate through the mesh and colonize the cages in high numbers, and thus provide the enclosed crabs with alternative sources of food (see [20] for details about the caging methods). Before adding the seeds in the cages, all resident epifauna and infauna were thoroughly removed using hand nets with 1 mm mesh. Sediment was sieved with the nets up to 5 cm depth. Crabs were collected within 1 hour before the beginning of each trial at the same depth and location as where the experiment was carried out. Carapace width and wet weight and were measured in each crab before and after the experiment to check for molt and assess the condition of the crabs after the enclosure period. Two trials of 1-week were carried out using 3 replicates of each treatment in each trial. At the beginning of each trial, 200 eelgrass seeds and 20 artificial seeds (mimics) were added to each plot. The trials were initiated during calm weather conditions and no transport of seeds were observed during the start of the trials.

Seed mimics were made from nylon cord and designed to mimic the size, shape and specific tissue density of natural eelgrass seeds (1.5 x 3.0 mm diameter; density of 1 g cm^−3^; see [41] for more details on the seed mimics). Pilot studies showed that seed mimics were not damaged or consumed by the assessed seed predators. Losses of seed mimics should therefore reflect seed transport and other non-predatory losses. Pilot tests were performed to calculate the minimum flow velocities necessary transport seeds of eelgrass and seeds mimics using a hydraulic flume. In the test, eelgrass seeds started to move at flow velocities higher than 14–16 cm/s while mimics moved at 10–12 cm/s over sandy sediment. Sinking velocities were also higher for eelgrass seeds (6–7 cm/s) than for seed mimics (4.5–5 cm/s). These differences in seed transport and sinking velocities indicate that seed mimics would be transported at slightly lower flows compared to natural seeds. Thus, using loss rates of mimics to approximate the loss of natural seed due to transport would result in a slight overestimate of the effect of water flow on the overall loss.

At the end of a 1-week trial, remaining seeds were collected by sieving (1 mm) the top 5–10 cm of the sediment from the plots, and counted in the laboratory. Sieving controls were performed to check the accuracy of the seed recovery method, which showed that 96% of the seeds were recovered (n = 5).

Rates of seed losses caused by predation and transport was estimated from the field experiments. First, seeds losses due to handling (not being able to recover all seeds) were estimated as the loss of eelgrass and artificial seeds found in the closed cages without crabs. Then, seed losses were estimated by subtracting the seed losses on each treatment to the seed losses on the close cage treatment, to remove the handling effect (Eq 1).

Seed losses=Seed loss "Treatment"−Seed loss "Cage Close" (1)

Seed losses due to predation were estimated by subtracting the losses of seeds mimics from the losses of eelgrass seeds. Seed losses due to transport were estimated as the losses of seed mimics. Losses of seed mimics due to transport in the caged treatments were assumed to be zero, and losses in the uncaged treatments (Open) were assumed to be a result only of transport.

### Seed predation in the field: 1-month and 8-month experiments

To assess the effect of seed predation and transport on seed recovery and seedling establishment over larger time scales, a 1-month and an 8-month experiment were carried out, respectively, in the Gullmars fjord area using similar methods as in the 1-week experiment. The 1-month experiment was performed from 6-Aug to 4-Sep of 2014 at the same location as the 1-week experiment using 3 different cage treatments: eelgrass seeds planted on the sediment surface (Open), covered with sand (Open+S) and protected with a closed cages (Cage C) using 200 eelgrass seeds and 20 seed mimics in each plot (n = 3). The main aim of this study was to estimate losses due to transport and predation in uncaged treatments over a 1 month period, by comparing losses of natural and mimic seeds, and if covering the seeds with sand would affect losses over 1 month period. The closed cage treatment was indented as a control, to estimate losses not due to transport and predation. Loss rates were calculated in the same way as explained for the 1-week experiment. Cage walls were cleaned weekly from fouling. A visual inspection at the end of the experiment did not show any change in the sediment type caused by the cages, which could have altered the treatment effect.

The 8-months experiment was performed from Sep 2013 to May 2014, which coincides with the dormancy and germination period of eelgrass seeds in the Swedish west coast [28]. In this experiment the aim was to assess the effect of predation on both seeds and possibly seedlings by assessing the rate of seedling establishment in the spring. The experiment was carried out at approximately 1.8 m depth on unvegetated sediment, approximately 20 m from a natural *Z*. *marina* meadow in a sheltered bay at the island Gåsö. In addition to the Open and Cage C treatments used in the previous experiment, an open cage treatment was added (Open C), with 15x15 cm openings at each side placed 3–5 cm above the sediment surface. These openings allowed predators such as shore crabs to access the cage [20], but reduced seeds from being transported out by hydrodynamics. One-month of continuous measurements of flow velocities at the study site in the fall using an acoustic Doppler velocimeter (Vector, Nortek), showed velocities (<10 cm/s) at the experimental site (E. Infantes, *unpubl*. *data*), which is below the threshold for transport of seeds (i.e. 14–16 cm/s), making seed transport out of the cages highly unlikely. Two hundred eelgrass seeds were placed on the sediment surface in each plot (n = 4). No seed mimics were used in this experiment. Since the Swedish winter is characterized by low water temperature and low light conditions [28], which reduces algae growth, cages were not cleaned during the winter. In the spring when the experiment was terminated, the cages were only lightly fouled with microalgae. Visual inspection of the sediment surface at the end of the experiment indicated slightly finer sediment inside the closed and open cages compared to plots without cages, but appeared not to differ between the two cage treatments.

### Statistical analysis

The effect of seed burial depth on predation in the laboratory study was assessed using a 2-way fixed factor ANOVA model with seed predator species and seed burial depth as independent variables and percent predation as the dependent variable. The effect of seed predation in the 3 cage experiments was assessed using 1-way fixed factor ANOVA models with cage treatments as independent variables and percent losses of eelgrass seeds and seed mimics as dependent variables. The loss of natural and seed mimics was analyzed in separate models to avoid pseudo-replication since they were present in the same plots. Before analyses were performed, all data were tested for homoscedasticity with Cochran’s C-test. The data from the laboratory study were square root-transformed to homogenize variances [42]. All figures show untransformed data. A posteriori multiple comparisons were carried out with the Student-Newman-Keuls (SNK) procedure.

## Results

### *Zostera marina* seed predators

**Fig 2:**
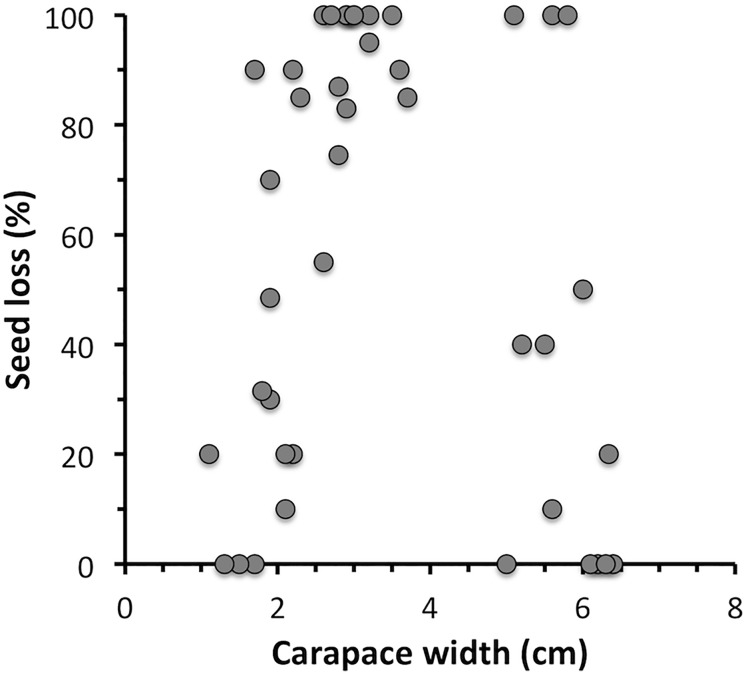
Size of shore crab (*C*. *maenas)* and predation of eelgrass seeds.

Three of the 9 assessed species repeatedly consumed or damaged eelgrass seeds in the laboratory. Shore crabs showed the highest predation rates with 51.5 ± 7.5% (mean ± SE) of the 10 eelgrass seeds consumed per day (Table 1). Shore crabs appeared to consume the whole seed as very little remains were found in the tanks. Predation was found in all tested size-classes, being very variable for the 11–64 mm carapace widths, CW, (Fig 2). The sea urchin *Strongylocentrotus droebachiensis* also showed high consumption rates of seeds (37% on average), whereas the hermit crab *Pagurus bernhardus* mainly damaged the seeds, as indicated by broken seed coats and seed embryos in the tanks, resulting in 6% loss of seeds on average. Predation rates in sea urchins and hermit crabs did not seem to be size related. No losses or damages of seeds were caused by the other species (Table 1).

### Seed burial depth and predation

**Fig 3:**
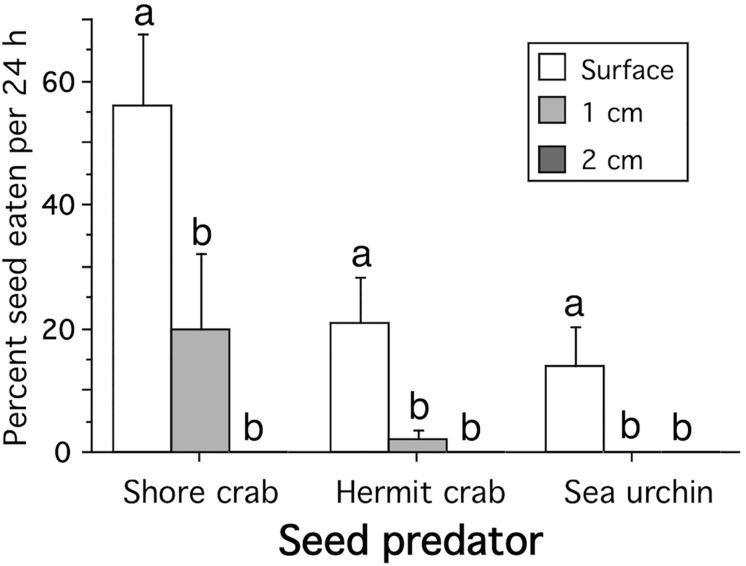
Seed burial depth and predation. Seeds consumed per day (mean+SE) by three potential seed predator species as a function of burial depth. Different letters above bars denote significant different means between sediment treatments (SNK-test at P<0.05).

Testing seed predation in more natural conditions with sediment, showed that burial depth decreased consumption rates significantly for all predators and that shore crabs consumed significantly more seeds (on average 30% per day) than hermit crabs and sea urchins (7.7 and 4.7%, respectively), which did not differ from each other (Table 2; SNK-test at p<0.05; Fig 3; S1 Table). Shore crabs consumed 56% of the seeds on the sediment surface, while the hermit crab *P*. *bernhardus* and the sea urchin *S*. *droebachiensis* consumed or damaged 21% and 13% of the seeds, respectively (Fig 3). Predation rates were significantly lower when seeds were buried at 1 cm depth, where shore crabs, hermit crabs and sea urchin consumed on average 20, 2 and 0% of the seeds, respectively. At 2 cm depth, seeds were not consumed by any species. The gastropod *Hinia nitida* did not prey on seeds, but buried 10.2 ± 3.1% (mean ± SE) of the seeds below the sediment surface. The seed burial depth caused by *H*. *nitida* was not assessed.

**Table 2:** Two-factor ANOVA model testing the proportion of seeds eaten (sqrt-transformed) as a function of seed predator (shore crab, hermit crabs and sea urchins) and burial depth (0, 1, and 2 cm).

	df	MS	*F*	*p*
**Seed predator (A)**	2	37.6	5.7	**0.0047**
**Burial depth (B)**	2	146.3	22.2	<**0.0001**
**A x B**	4	11.5	1.8	0.1457
**Residual**	86	6.6		

### Seed predation in the field: 1-week experiment

**Fig 4:**
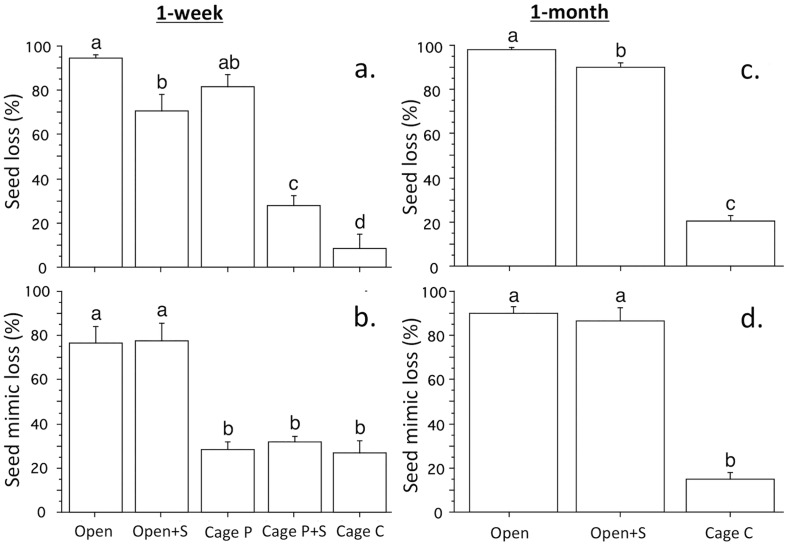
One-week and one-month cage experiment. Loss of (a) eelgrass seeds and (b) seed mimics in the one-week, and loss of (c) eelgrass seeds and (d) seed mimics in the one-week. Seeds with: no cage (Open), no cage + sand cover (Open+S), cage + shore crab (Cage P), cage + shore crab + sand cover (Cage P+S) and cage (Cage C). Different letters above bars denote significant different means (SNK-test at P<0.05). 200 eelgrass seeds and 20 seed mimics used per experimental. Mean+SE.

Losses of natural eelgrass seeds in the 1-week field experiment differed significantly between cage treatments, indicating strong effects of both seed predation and transport, and a reduction of predation by covering the seeds with sand (Table 3, S2 Table). In the closed cage treatments, loss rates in cages with one enclosed shore crab (on average 82%) was significantly higher than when the seeds were covered with sand, and in caged without crabs (on average 28 and 8% loss, respectively; SNK-test at p<0.05; Fig 4a). Losses of seed mimics in the closed cage treatments showed a strikingly different pattern with relatively low and similar losses (27–32%) that did not differ significantly between treatments (Fig 4b). Losses of natural seeds placed on the sediment surface without cages were high (on average 95%) and similar to losses in the cage treatment with crabs, but losses were significantly lower in uncaged treatments where the seeds were covered with sand (on average 71%; Fig 4a). Losses of seed mimics were significantly higher outside the cages (77–78%), but were not significantly affected by sand cover (Fig 4b). The highest percentage of seed coats (12%), which indicate that seeds have been subjected to predation, were found in cages with shore crabs and seeds on the sediment surface. In the rest of the treatments, the percentage of seed coats was <2%.

**Table 3:** Cage experiments. One-way ANOVA models testing the effect of cage treatments on the loss of natural and seed mimics in 1-week, 1-month and 8-month long field experiments.

		Eelgrass seeds			Seeds mimics		
	df	MS	*F*	*p*	MS	*F*	*p*
**1-Week**	4	8122.1	45.6	<**0.0001**	4200.8	20.1	**<0.0001**
	25	177.9	---	---	308.8	---	---
**1-Month**	2	5509.7	457.0	<**0.0001**	5386.1	102.1	**<0.0001**
	6	12.0	---	---	52.7	---	---
**8-Months**	2	261.3	336.0	<**0.0001**	---	---	---
	9	0.7	---	---	---	---	---

The estimated predation rates on seeds on the sediment surface in the closed cages and open plots was on average 73% and 36% per week, respectively, after excluding losses found in the closed cages that prevented seed transport and predation (Table 4). Sand cover reduced the estimated predation rates with on average 73% and 68%, respectively. The estimated loss of seed mimics due to transport was 50% per week, on average.

**Table 4:** Estimates of seed losses in the 1-week and 1-month experiments. Percent loss of eelgrass seeds and mimics in all treatments. Seed losses were estimated by subtracting the losses on each treatment to the losses on the Cage C (to exclude the handling effect, Eq 1). Losses by transport were estimated as the losses of mimics. Losses by predation were estimated as the difference between losses by transport and losses excluding handling. Mean and (Std. Err).

		All seed losses		Losses excl. handling		Transport losses	Predation losses
		Eelgrass	Mimics	Eelgrass	Mimics		
**1-Week**	**Cage C**	8.4 (6.6)	26.7 (5.9)	0	0	0	0
	**Cage P+S**	28.1 (4.3)	31.7 (2.8)	19.7	5.0	0	19.7
	**Cage P**	81.7 (5.6)	28.3 (3.7)	73.3	1.6	0	73.3
	**Open S**	70.8 (7.2)	77.5 (8.1)	62.4	50.8	50.8	11.6
	**Open**	94.7 (1.4)	76.7 (7.4)	86.3	50.0	50.0	36.3
**1-Month**	**Cage C**	20.2 (2.9)	15.0 (2.9)	0	0	0	0
	**Open S**	90.2 (1.8)	86.7 (6.0)	70	71.7	71.7	1.7
	**Open**	98.0 (0.8)	90.0 (2.9)	78	75	75	3

### Seed predation in the field: 1 and 8 month experiments

The result in the one-month cage experiment was similar to the one-week study, although loss rates were over all higher. Losses of natural seeds planted on the sediment surface without cages (on average 98%) were significantly higher than for seeds covered with sand (90%) and seeds placed in cages (20%; SNK-test at p<0.05; Fig 4c, S3 Table). In comparison to natural seeds, loss of seed mimics planted on the sediment surface was slightly lower (on average 90%) and did not differ significantly from the losses of mimics overed with sand (87%). Losses of mimics in cages were significantly lower (on average 15%; SNK-test at p<0.05; Fig 4d). After excluding losses in the closed cages, most remaining losses (78% per month) was estimated to be caused by seed transport (75%).

**Fig 5:**
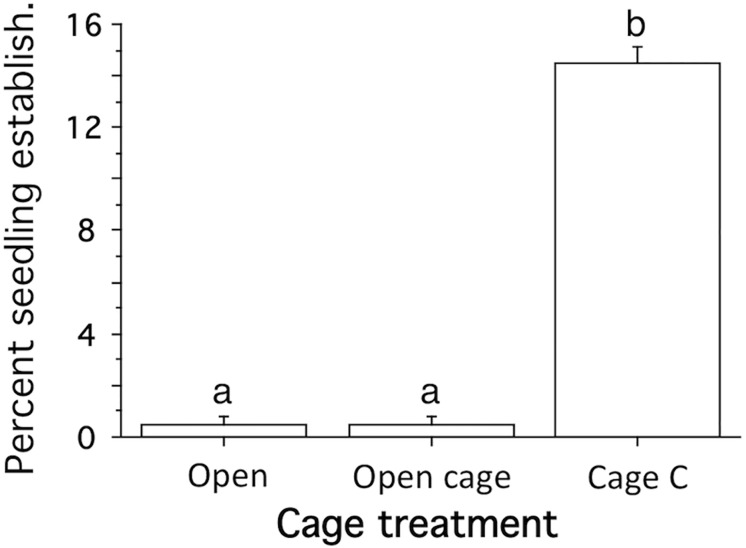
Eight-month cage experiment: Seedling establishment. Seeds with no cage (Open), cage with small holes that let predators in, but prevent seed transport by hydrodynamics (Open Cage) and seeds protected with cages (Cage C). Different letters above bars denote significant different means (SNK-test at P<0.05). 200 seeds planted per plot. Mean+SE.

In the 8-month experiment, the percent seedling establishment rate (no. seedlings/no. of seeds planted) was significantly higher when seeds were protected by closed cages (14% seedlings) compared to cages that allowed predator access, but reduced transport of seeds by hydrodynamics (0.5% seedling) and seeds planted without cages (0.5% seedlings; SNK-test at p<0.05; Fig 5; S4 Table).

## Discussion

This study identifies three previously undescribed predators of eelgrass seeds, and presents evidence that seed-predation can constitute a major source of seed-loss. We suggest that seed-predation from shore crabs may play a key role in the present lack of natural recovery of eelgrass along the Swedish northwest coast, that will challenge restoration attempts in the area. Our results demonstrate that burying seeds at 2 cm below the sediment surface will decrease predation rates and could improve chances of successful restoration of eelgrass.

### *Z*. *marina* seed predators

The laboratory studies found that shore crabs *C*. *maenas*, the sea urchin *S*. *droebachiensis* and the hermit crab *P*. *bernhardus* could consume significant amounts of eelgrass seeds, where predation rates of the shore crab was about twice as high as of the other predators. Previous studies have shown that macroinvertebrates, principally decapod crustaceans, are key predators on seagrass seeds [30,33,43–46], although fish, reptiles and waterfowl have also been reported to ingest seeds of seagrass [32,47], see Table 5. This study shows for the first time that also an echinoderm can prey on eelgrass seeds.

**Table 5:** Summary of species that prey on *Z*. *marina* seeds. nd = no data, b = biomass (g) and b = head width.

	Animal	Mean size (mm)	Type	Effect	Mean predation (%)	Reference
**Crustacean**	*Ovalipes ocellatus*	18	Seeds	Damaged/Consumed	93	*Wigand & Churchill 1988*
	*Pagarus longicarpus*	9	Seeds	Consumed	73	*Wigand & Churchill 1988*
	*Panopeous herbstii*	22	Seeds	Consumed	63	*Wigand & Churchill 1988*
	*Carcinus maenas*	10–65	Seeds	Consumed	52	This study
	*Zeuxo sp*.	1–4.5	Seeds	Damaged/Consumed	14	*Nakaoka 2002*
	*Pagarus bernhardus*	12	Seeds	Damaged/Consumed	6	This study
	*Pagarus longicarpus*	7	Seedlings	Damaged/Consumed	93	*Wigand & Churchill 1988*
**Mollusk**	*Ilyanassa obsoleta*	20	Seedlings	Damaged/Consumed	47	*Wigand & Churchill 1988*
	*Littorina littorea*	20	Seedlings	Damaged/Consumed	10	*Wigand & Churchill 1988*
**Echinoderm**	*Strongylocentrotus droebachiensis*	22	Seeds	Consumed	37	This study
**Fish**	*Fundulus heteroclitus*	51	Seeds	Consumed	5	*Wigand & Churchill 1988*
	*Fundulus heteroclitus*	84	Seeds	Consumed/Dispersed	nd	*Sumoski & Orth 2012*
	*Fundulus majalis*	62	Seeds	Consumed	1	*Wigand & Churchill 1988*
	*Opsanus tau*	36	Seeds	Consumed	2	*Wigand & Churchill 1988*
	Sphoeroides maculatus	143	Seeds	Consumed/Dispersed	nd	*Sumoski & Orth 2012*
	Lagodon rhomboides	123	Seeds	Consumed/Dispersed	nd	*Sumoski & Orth 2012*
**Worm**	*Arenicola marina*	1-10^a^	Seed	Burial	nd	*Valdemarsen et al 2011*
**Reptail**	Malaclemys terrapin	15-30^b^	Seeds	Consumed/Dispersed	nd	*Tulipani & Lipcius 2014*
	Malaclemys terrapin	nd	Seeds	Consumed/Dispersed	nd	*Sumoski & Orth 2012*
**Bird**	Aythya affinis	nd	Seeds	Consumed/Dispersed	nd	*Sumoski & Orth 2012*

The laboratory studies demonstrated that shore crabs 11–64 mm CW could consume high numbers of eelgrass seeds (Fig 2). Since alternative prey was not available, and the crabs were confined to small containers in the laboratory study, these consumption rates should be viewed with caution. However, in the field experiment natural prey sources were available in the sediment for the enclosed crabs, and small epibenthic fauna could also enter through the 1 mm mesh of the cage. Predation rates in the cage experiment should therefore better represent natural rates. Using data from the 1-week cage experiment, and excluding losses of seeds found in the closed cages without predators, it was estimated that one enclosed shore crab consumed 147 seeds on average, causing a 73% loss of seeds during the 1-week experiment (equivalent to 21 seeds crab^-1^ per day). These high consumption rates, despite availability of alternative prey, suggest that eelgrass seeds constitute an attractive prey item for shore crabs. Eelgrass seeds contain a high concentration of starch [48], which may constitute an important source of energy for the crabs. However, although alternative prey sources were available to the enclosed crabs, the amount was never assessed. It is therefore possible that low abundance of alternative prey sources could have resulted in unnatural high predation rates of seeds by the enclosed crabs. To obtain more accurate estimates of the predation pressure on seeds, further studies, including food-choice experiments, are needed that assess consumption rates of seeds when presented together with controlled amounts of natural alternative food sources.

The shore crab is an opportunistic omnivore that feeds on various benthic organisms. Although it prefers molluscs, crustaceans and polychaetes, it may also consume plant material and detritus [33,35,49]. Recent studies in Canada indicate that shore crabs may graze on the basal meristem of eelgrass shoots where the tissues are younger and softer [50]. In addition, *C*. *maenas* can damage 39% of eelgrass shoots transplanted in tanks [49] and have been related to the decline of natural eelgrass beds [51] but their impact on seed predation has not been assessed. The present study is, as far as we know, the first to demonstrate shore crabs also consume eelgrass seeds.

### Importance of seed predation

Earlier studies of seed planting along the Swedish northwest coast have found very high loss rates of seeds, resulting in seedling establishment rates <1% on average. A study assessing seed planting methods found that the seedling establishment rates increased 2–6 times if seeds were covered with a 2 cm layer of sand [28]. However, it was not clear to what degree seed predation was responsible for those observations since seed and seedlings could also be lost by being transported by waves and currents [28]. Results from the present study suggest that seed predation is a major factor behind the unusual high loss rates of seeds in Swedish waters.

In shallow habitats, sediment dynamics and transport of seeds by hydrodynamics may cause very high loss rates of eelgrass seeds [52]. This was supported in the present study in the more exposed bay where 77% and 90% of the seed mimics were lost in the open plots after one week and one month, respectively, likely a result of seed transport by waves and currents. In the one-week experiment, it was estimated (after excluding losses found in cages that prevented seed transport and predation) that 50% of the loss of natural seeds in the open plots were due to hydrodynamic transport and 36% was caused by seed-predation. However, these estimates likely represent an overestimate of seed transport, and consequently an underestimate of seed predation, because the transport estimates were based on losses of seed mimics, which were transported at approximately 27% lower flow velocities than were natural seeds, according to the laboratory studies. The effect of seed predation could also be hidden by transport of seed mimics accumulating over entire experimental period, as indicated by the low estimate of seed predation in the one-month cage experiment. Most of the seed predation probably occurred in the beginning of the experiment when high seed densities could attract opportunistic predators such as shore crabs that display a type III functional response to prey densities [53]. In contrast, seed transport likely occurred later in the experiments since the trials were initiated during calm weather conditions when no seed transport was observed. Since the natural densities of shore crabs that can consume eelgrass seeds in shallow areas in the study area are similar to the densities enclosed in the cages (5 crabs m^-2^) [39,40], natural predation rates of seeds may be as high as measured in cages with crabs (over 70% per week). High seed predation in the non-cage treatments was also supported by approximately 70% lower loss of natural seeds covered with sediment, whereas sand treatment did not affect losses of seed mimics (Fig 4). This result also suggests that covering the seeds with sand mainly prevent predation (and not seed transport), and the positive effect of seed burial on seedling establishment found in earlier studies [28] also reflected high rates of predation. The importance of seed predation is also supported by the 8-month study carried out in a more sheltered bay. Here, cage treatments were used to separate predation and transport effects, showing a much higher seedling establishment rate when predators were excluded (14%) compared to when predator had access to the seeds (0.5%). No difference in seedling establishment rate between open and no cage treatments suggests negligible effects of seed transport by hydrodynamics, despite an indicating of higher sedimentation rates and lower flows in the open cage treatment.

These results provide strong support that predation is a major source of seed loss and that shore crabs are the dominant seed predator in the study area. Shore crabs are not only efficient seed predators (as discussed above), but also abundant in eelgrass habitats in the Swedish west coast (4–20 shore crabs >10 mm m^-2^) [20,22,40]. The high predation rate and density of shore crabs would cause a substantial reduction of the eelgrass seed production. Densities of reproductive shoots along the Swedish northwest coast are relatively low (on average 6–10 shoots m^-2^) where each shoot produces on average around 40–60 viable seeds (E. Infantes, unpubl. data). Thus, the annual production of seeds in Swedish eelgrass beds is only in the order of 240–600 seed m^-2^, which one crab could consume in 15–30 days, according to this study.

The importance of sexual reproduction for the persistence and growth of eelgrass populations in Scandinavian waters is not well known. One study from the Swedish northwest coast found that 35% of young eelgrass shoots in the spring originated from seeds [54], and a recent genetic study in the Gullmarsfjord area estimated the linear clone size within meadows to 2–10 m [27], indicating that sexual reproduction constitutes a significant part of the annual growth in eelgrass meadows. Seed predation could therefore have a large impact on the population dynamics of eelgrass in the study area. However, it is important to note that the field studies were performed outside natural eelgrass meadows, and that the seed predation rates of shore crabs may differ inside the meadow. The results from this study may therefore be more relevant for effect of seed predation during natural colonization or restoration with seeds of unvegetated areas. The impact of predation may be particularly large during colonization by rafting reproductive shoots, when seeds are critical for establishment and growth of new patches. It is, therefore, likely that seed predation has played an important role by preventing natural re-colonization along the Swedish northwest coast, and could present a challenge for restoration efforts, particularly when seeds are used.

### Seed burial and implications for restoration

In the laboratory, seeds buried at 1 cm depth were consumed significantly less than those at the sediment surface, while seeds at 2 cm sediment depth were not consumed by any of the focal predator species. Seeds planted at 2 cm depth have shown higher germination and seedling survival than at deeper depths or at the sediment surface [28,55–57]. Seeds can be naturally buried in the sediment by processes such as sediment dynamics driven by hydrodynamics and sediment reworking by the lugworm *Arenicola marina* [41,52]. In this study, *Hinia nitida* was found to bury 10% of seeds below the sediment surface, which could increase the survival and success of seedlings. It is the first time that seed burial by *H*. *nitida* has been described. In addition, a sediment layer might also have additional effects such as preventing the development of marine phytophthora species [58].

During the site-selection process for restoration, water quality, light, hydrodynamics exposure, depth, sediment type are some of the main factors assessed [59]. Results of the present study underscore the major influence that predators, in particular *C*. *maenas*, can have on seed survival and the need for their consideration during restoration since they could have a large impact on the restoration success. New methods could be developed to reduce seed predation by burying seeds in the sediment and minimize the effect of these species. For example, mechanical planting machines to bury the seeds could be applied to reduce predation [60,61]. In addition, planting a high number of seeds over large spatial scales may also increase the chances of survival by temporarily overwhelm seed predators. This is partly supported by a recent review of restoration studies that found higher survival and growth of plants when a higher number of shoots/seeds were planted [62].

### Implications for regime shifts in coastal ecosystems

The large impact of predation from shore cabs on eelgrass seeds may have implications for the large-scale shift in macro-vegetation that has occurred along the Swedish northwest coast. Since the 1980s, mats of filamentous macroalgae have increased dramatically in shallow coastal areas and at the same time, more than 60% of eelgrass has vanished [19,23,63]. Both nutrient pollution and overfishing are considered the main reasons behind this change. The loss of large predators in the coastal ecosystem is thought to have caused a trophic cascade, releasing filamentous macroalgae from grazing control, which can bloom and form large mats at high nutrient conditions in the absence of functional grazers, with negative effects on eelgrass growth [19–21,37,64].

**Fig 6:**
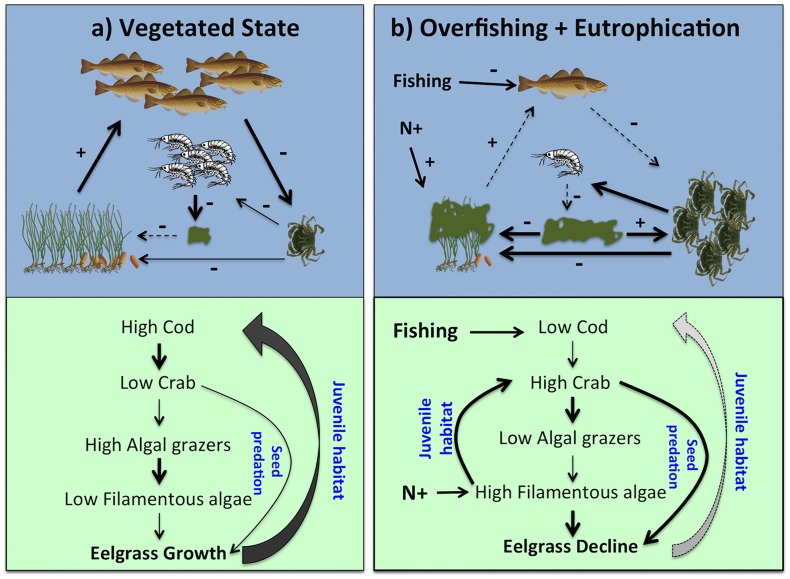
Regime shifts and positive feedback mechanism in eelgrass systems involving seed predation by shore crabs. Arrow thickness indicates the strength of the trophic interaction and habitat effects, where dashed lines denote weak effects. Plus and minus signs indicate positive and negative effects, respectively. In the vegetated state (a), with little impact of nutrient pollution and overfishing, eelgrass beds provide nursery habitats for a large cod population that can control the abundance of shore crabs and other small predators. The low abundance of mesopredators allows algal grazers to control ephemeral algae from overgrowing eelgrass and prevents high rates of seed predation from crabs. In the overfished and eutrohicated state (b), overfishing of cod has released shore crabs and other mesopredators from predation control, increasing predation on seeds. The high predation rate on algal grazers releases ephemeral algae from grazer control than can bloom in the nutrient rich water with negative effects on eelgrass, and indirectly in cod recruitment. The high abundance of ephemeral algae increases recruitment of shore crabs by increasing availability of shelter for juvenile crabs.

Shore crabs may play a key role in the loss and lack of recovery of eelgrass along the Swedish northwest coast by being promoted both by overfishing and eutrophication, and by causing both direct and indirect negative effects on eelgrass (Fig 6). Shore crabs are a major food source for Atlantic cod in coastal areas, and cod biomass has been negatively correlated with the abundance of shore crabs, which has increased 2–3 times along the Swedish west coast since the 1970s [65]. In addition, abundance of shore crabs correlates positively with the abundance of filamentous algal mats along the Swedish west coast [66]. Studies have demonstrated that filamentous macroalgae constitute excellent nursery habitats for shore crabs by decreasing predation mortality and increasing the juvenile crabs recruitment in the study area [40,53,67].

The present study suggests that a high abundance of shore crabs may not only affect eelgrass on a negative scale indirectly through reduced predation on algal grazers, but also directly through high predation on eelgrass seeds. In addition, shore crabs may cause negative effects by consuming and uprooting eelgrass shoots [36,49–51]. Shore crabs may therefore act as a strong feedback mechanism that maintains a state dominated by algal mats, by decreasing the nursery habitat of its dominant predator (cod) and also by promoting the growth of its own nursery habitat (algal mats; Fig 6b). These two feedback loops may cause an accelerating loss of eelgrass and be partly responsible for the regime shift observed in the study region. As far as we know, this is the first time a possible double feedback mechanism has been identified in a marine system that involves the same trophic interactions. To overcome these problems and shift the system back to a state dominated by eelgrass, multiple measures would be required that would allow the return of large fish predators to the coastal ecosystems, likely involving both increased regulation of fishing and nutrient pollution, as well as restoration of eelgrass.

## Supporting Information

S1 Table Raw data of burial depth experiment. (XLSX)

S2 Table Raw data of 1-week experiment. (XLSX)

S3 Table Raw data of 1-month experiment. (XLSX)

S4 Table Raw data of 8-month experiment. (XLSX)
